# Mechanisms of HAHV-1 Interaction with Hemocytes in *Haliotis diversicolor supertexta*: An *In Vitro* Study

**DOI:** 10.3390/biology14020121

**Published:** 2025-01-24

**Authors:** Mao-Le Wei, Ya-Nan Li, Jing-Li Wang, Cui-Ping Ma, Hui-Gang Kang, Pei-Jun Li, Xiang Zhang, Bo-Wen Huang, Chang-Ming Bai

**Affiliations:** 1 Sino-UAE International Cooperative Joint Laboratory of Pathogenic Microorganism Rapid Detection, Qingdao Nucleic Acid Rapid Detection Engineering Research Center, Qingdao Key Laboratory of Nucleic Acid Rapid Detection, College of Biological Engineering, Qingdao University of Science and Technology, Qingdao 266042, China; liuli000315@gmail.com (M.-L.W.); wjl19863763762@163.com (J.-L.W.); mcp169@163.com (C.-P.M.); kanghuigang0123@163.com (H.-G.K.); 2 State Key Laboratory of Mariculture Biobreeding and Sustainable Goods, Key Laboratory of Maricultural Organism Disease Control, Ministry of Agriculture, Yellow Sea Fisheries Research Institute, Chinese Academy of Fishery Sciences, Qingdao 266071, China; liyn@yctu.edu.cn (Y.-N.L.); 17622735933@163.com (P.-J.L.); zhxiang1997@126.com (X.Z.); 3 College of Ocean and Biology Engineering, Yancheng Teachers University, Yancheng 224007, China; 4 Laboratory for Marine Fisheries Science and Food Production Processes, Qingdao Marine Science and Technology Center, Qingdao 266237, China; 5 Shandong Center of Technology Innovation for Oyster Seed Industry, Qingdao 266105, China

**Keywords:** HAHV-1, abalone, hemocytes, transcriptome, pathogen−host interaction

## Abstract

Haliotid herpesvirus 1 (HAHV-1) causes significant damage to the abalone aquaculture industry. Knowledge of HAHV-1 invasion and host defense mechanisms is limited due to the lack of stable molluscan cell lines. The present study established an *in vitro* infection model of HAHV-1 using the primary suspension cultures of hemocytes from *Haliotis diversicolor supertexta* and *Haliotis discus hannai*. The cytopathic effects of HAHV-1 on adherent-cultured hemocytes of both species were also investigated. The HAHV-1 DNA loads were firstly monitored by means of quantitative PCR during the development of viral infection, and subsequently the mechanism of interaction between HAHV-1 and hemocytes was explored by means of a transcriptome analysis. *H. diversicolor supertexta* hemocytes exhibited a high degree of susceptibility to HAHV-1, with viral loads reaching a peak of 4.0 × 10⁷ copies/ng DNA. In contrast, no significant replication was observed in *H. discus hannai* hemocytes. Transcriptome analysis revealed that HAHV-1 evades the host immune response in the early stages of infection, and hijacks the host’s energy and redox metabolism to promote its replication at the late stages. Consequently, this study provides a valuable reference point for the investigation of virus−host interaction between HAHV-1 and abalone *in vitro*.

## Simple Summary

Abalone are high-priced and mobile-grazing gastropod mollusks, cultivated or fished primarily in east Asia and Australia. A devastating pandemic related to Haliotid herpesvirus 1 (HAHV-1) swept across mainland China, Taiwan, and Australia, causing severe damage and property loss to the industry. The pandemic has led to the collapse of the *Haliotis diversicolor supertexta* aquaculture industry in mainland China. The mechanisms of interaction between HAHV-1 and susceptible abalone remain underexplored. In this study, we establish an *in vitro* infection model for HAHV-1 and investigate the virus–host interaction mechanisms. The results indicated that the virus hijacks the host’s actin cytoskeletons and microtubule network, resulting in immune deficiency during the early stages of infection. Furthermore, the virus hijacks the host’s energy metabolism and redox metabolism, contributing to the rapid increase of viral loads in the later stages of infection. These findings provide valuable insights into the pathogenic mechanism of HAHV-1.

## 1. Introduction

Since its initial identification in 1999 in Dongshan County, Fujian Province, China, HAHV-1 has resulted in substantial damage to the global abalone farming industry and wild abalone populations, with China’s *Haliotis diversicolor supertexta* aquaculture being particularly impacted, leading to its collapse [1,2]. Outbreaks of HAHV-1 occur mainly in coastal areas of southern China and Australia, and phylogenetic analyses reveal a high degree of genetic similarity among HAHV-1 variants from mainland China, Taiwan, and Australia [3,4,5]. The main susceptible hosts are *H. diversicolor supertexta*, *Haliotis rubra*, *Haliotis laevigata*, *Haliotis conicopora* and their hybrids, while *Haliotis iris* and *Haliotis discus hannai* are not susceptible to HAHV-1 [1,6]. Infection with HAHV-1 leads to two distinct modes of mortality: acute and chronic, both of which involve viral infection of neural tissue and hemocytes, respectively. The acute form is marked by nervous tissue necrosis, whereas the chronic form is characterized by hemocyte infiltration [4,7,8]. Although the complete genome of HAHV-1 has been sequenced, it is evolutionarily distant from vertebrate herpesviruses, which complicates the prediction of its protein functions through homology comparison, resulting in most of its predicted proteins remaining unidentified [9,10,11]. Therefore, the molecular mechanisms underlying HAHV-1−host interactions, particularly at the protein level, remain to be fully elucidated [12,13,14].

The development of an *in vitro* mollusk infection platform is crucial to the study and management of molluscan viruses, as it provides a controlled, standardized, and reproducible experimental platform for viral research [15]. The absence of molluscan cell lines has impeded the application of conventional viral research methods to the study of HAHV-1 [12]. The main challenges in establishing molluscan cell lines include simulating complex natural environments and preventing contamination by marine microorganisms [15,16,17]. Researchers have made numerous attempts to establish cell lines in mollusks using tissues such as the gills, mantle, and heart isolated from *Haliotis midae* [18], *Chlamys farreri* [19], *Ruditapes decussatus* [20] and *Crassostrea gigas* [15,17,21]. However, these attempts have not yet achieved stable continuous passage. Hemocytes represent the primary immune effector cells in mollusks, playing a crucial role in exogenous invading pathogen invasion and defense. These cells are involved in various innate immune processes such as phagocytosis, inflammation, and the secretion of antimicrobial peptides [22,23]. A taxonomic classification of molluscan hemocytes is typically based on their morphological characteristics, leading to the identification of two primary types: granulocytes, which are characterized by the presence of abundant cytoplasmic granules, and hyalinocytes, which exhibit a paucity of granules or the absence thereof. Granulocytes primarily play a phagocytic role in the immune process of mollusks, exhibiting strong phagocytic capabilities against a variety of pathogens and foreign particles. In contrast, the phagocytic ability of hyalinocytes is relatively weak. These two cell types work together in the organism’s immune defense [24,25]. Consequently, hemocytes have emerged as a compelling *in vitro* model to elucidate the mechanisms underlying HaHV–host interactions. Hemocytes are more easily obtained than other tissue cells, and their cultivation conditions are relatively less complex to manage and control [16]. An increasing number of *in vitro* models of molluscan hemocyte infection are being used to elucidate the mechanisms of viral infection and host immunity [17,19,26].

The advancement of high-throughput sequencing technologies has engendered novel approaches to the study of the immune mechanisms in non-model organisms. In our previous studies, we conducted *in vivo* infection experiments with HAHV-1 on *H. diversicolor supertexta*, and transcriptomic analysis revealed a distinct delayed immune response. During the early stages of infection, there was a steady increase in HAHV-1 copies, but there were no significant alterations in the expression of immune-related genes. Conversely, in the later stages of infection, immune-related genes were overexpressed, indicating that during the early phase of infection, the virus can replicate without activating the host’s immune response. This finding is consistent with the hypothesis that the virus employs efficient immune evasion strategies [27].

In this study, an *in vitro* infection model for HAHV-1 in mollusks was established to further elucidate the interaction mechanisms between HAHV-1 and its host. The *in vitro* hemocyte infection test is conducted under controlled laboratory conditions, thus minimizing the influence of environmental factors and yielding more accurate results. Subsequently, we performed transcriptome sequencing on the suspension-cultured hemocytes of *H. diversicolor supertexta* infected at 24 and 60 h post-inoculation (hpi), and conducted a preliminary investigation of the interactions between HAHV-1 and *H. diversicolor supertexta* hemocytes based on a comprehensive analysis of KEGG and GO. The results obtained from this study laid the foundation for the development of an *in vitro* HAHV-1 infection platform and provided transcriptome data that can serve as a valuable reference point for the study of the interaction mechanisms between abalone and HAHV-1.

## 2. Materials and Methods

### 2.1. Animals and Experimental Infection

*H. diversicolor supertexta* (shell length range 5.23–6.64 cm, *n* = 30) was purchased from Xiamen, Fujian Province, China, and *H. discus hannai* (shell length range 6.56–7.77 cm, *n* = 30) was purchased from Weihai, Shandong Province, China, with 50 abalones of each species. After transfer to the aquaculture facility of the Yellow Sea Fisheries Research Institute (YSFRI), they were cultivated in 50 L tanks (25 pieces per tank) containing aerated, sand-filtered seawater for a period of two weeks. During this period, the salinity and temperature of the seawater were maintained at 30 ± 1 ppt and 18 ± 1 °C, respectively. During the acclimatization period, half of the seawater was replaced daily, and fresh seaweed (*Laminaria japonica*) was replaced daily. After the acclimatization period, 15 individuals of each species were randomly selected for HAHV-1 testing, which yielded negative results. All methods were conducted in accordance with the approved protocols and relevant guidelines.

### 2.2. Hemolymph Collection

Thirty individuals of *H. diversicolor supertexta* and *H. discus hannai* were selected, each in optimal condition and exhibiting robust health following a temporary rearing period. A 5 mL syringe fitted with a 23 G needle was employed to withdraw hemolymph from the cephalic arterial sinus of the abalone, with approximately 1 mL of fluid obtained from each specimen. The hemolymph from every ten abalones was pooled into a single sample, with three biological samples (replicates) from thirty abalones per species. The hemolymph was subsequently diluted with filtered sterile seawater and adjusted to a concentration of 1 × 10⁶ cells mL⁻¹. The hemocytes were placed on ice to prevent coagulation.

### 2.3. Morphologic Observation on Hemocytes

The extracted *H. diversicolor supertexta* and *H. discus hannai* hemolymph were inoculated into 12-well cell culture plates (LABSELECT, Shanghai, China) at 500 μL (1 × 10^6^ cells mL^−1^) per well. Following a 24 h culture period, the cells were fixed with 0.4% paraformaldehyde (Sagon Botech, Shanghai, China) for 15 min. Thereafter, the cells were washed twice in phosphate-buffered saline (pH 7.4) and then stained with Giemsa staining solution (Beyotime Biotechnology, Shanghai, China). Following the completion of the staining process, the hemocytes were examined and photographed using a Nikon Eclipse E80i light microscope (Nikon, Tokyo, Japan) [19].

### 2.4. HAHV-1 Suspension Preparation

The standard method for producing Ostreid herpesvirus 1 (OsHV-1) suspension was employed for the production of HAHV-1 viral inoculum. However, the dilution step utilized filtered sterile artificial seawater instead of natural seawater [28]. Infected with high HAHV-1 loads, *H. diversicolor supertexta* collected during abnormal mortality outbreaks in Guangdong Province, China, in 2003 were used to prepare viral inoculum. Negative tissue homogenates were prepared from *H. diversicolor supertexta* that had not been infected with HAHV-1, using the same methodology as that employed for the HAHV-1 suspension. Subsequently, 200 μL of each homogenate was extracted for the purpose of quantifying HAHV-1 (*n* = 3).

### 2.5. Challenge of Primary Cultured Hemocytes with HAHV-1

The challenge experiments were divided into adherent and suspension culture infection. The adherent culture group was used to observe cytopathic effects, and the suspension culture group was used to detect viral load changes and to perform transcriptome sequencing. All experiments were conducted in triplicate. Negative controls were processed alongside each treatment group, with all control conditions treated under the same experimental conditions to ensure consistency.

For the adherent culture infection, 500 μL of extracted hemolymph (1 × 10^6^ cells mL^−1^) was spread flat in a 12-well cell culture plate, and 1 mL of modified L−15 medium was added to each well. The L−15 medium was modified by the addition of 20.2 g/L NaCl, 0.54 g/L KCl, 0.6 g/L CaCl_2_,1 g/L MgSO_4_, 3.9 g/L MgCl_2_, 2 mmol^−1^ glutamine (Beyotime Biotechnology, Shanghai, China), 100 U mL^−1^ penicillin (TransGen Biotech, Beijing, China), 100 µg mL^−1^ streptomycin (TransGen Biotech), and 5% FBS (Beyotime Biotechnology). After static incubation at 18 °C for about 12 h, 10 μL of HAHV-1 suspension (10^5^ HAHV-1 copies/µL) was added to the challenge group, while the control group was administered an equal volume of negative tissue homogenate, and the culture medium was replaced every 24 h [19]. Cytopathic effects in hemocytes were observed using a Nikon Eclipse E80i light microscope (Nikon), and images were captured.

For the suspension culture infection, 500 μL of extracted hemolymph (1 × 10^6^ cells mL^−1^) was added to 1.5 mL tubes, which had been supplemented with 100 U mL^−1^ penicillin and 100 µg mL^−1^ streptomycin (TransGen Biotech). In the challenge group, 250 μL of HAHV-1 suspension (10^5^ HAHV-1 copies/µL) was added to each tube, and an equal amount of negative tissue homogenate was added to the control group. The culture was then incubated at 18 °C with slow agitation to keep the hemocytes in suspension [26].

### 2.6. DNA Extraction and HAHV-1 Quantification by qRT-PCR

Samples were collected at 0, 2, 4, 8, 18, 24, 48, 60, and 72 hpi in the suspension culture challenge group and control group, respectively, with three replicates per group. The collected hemolymph was then subjected to immediate centrifugation at 4 °C and 800× *g* for 8 min. The supernatant was the hemolymph and the precipitate was the hemocytes, which were stored at −80 °C in a cold refrigerator for reserve use. HAHV-1 DNA was extracted using a Roche High Pure Viral Nucleic Acid Kit (Roche Diagnostics, Risch-Rotkreuz, Swiss) according to the manufacturer’s protocol, and HAHV-1 DNA was quantified by qPCR against ORF 66 according to the protocol adapted from the World Organization for Animal Health (WOAH) Manual of Diagnostic Tests for Aquatic Animals, 2017, described in detail by Bai et al. [1]. We estimated the HAHV-1 infection burden of each sample as the mean genomic equivalent (GE) score (ng^−1^ of total DNA) for the three replicates.

### 2.7. RNA Extraction, cDNA Synthesis and Sequencing

Given the potential for hemocyte status to fluctuate over time in an *in vitro* setting, it is more appropriate to select viral challenge and negative control hemocytes that have been cultured for an equivalent duration for comparison. HAHV-1 challenge and negative control *H. diversicolor supertexta* hemocyte samples collected at 24 and 60 hpi were selected for transcriptome sequencing based on the trend of measured viral loads (*n* = 3). These 12 samples were designated as ZP24 (1–3), ZN24 (1–3), ZP60 (1–3), and ZN60 (1–3). Total RNA was extracted using TRNzol Universal Reagent (Tiangen Biotech, Beijing, China) according to the manufacturer’s instructions. RNA degradation and contamination were monitored on 1% agarose gels, and RNA purity was checked using a Nanodrop 2000 spectrophotometer (Thermo Fischer Scientific, Waltham, MA, USA). The RNA samples were subjected to testing and subsequently dispatched to Novogene Technology Co. Ltd. (Beijing, China) for high-throughput sequencing using the Illumina HiSeq platform (Illumina Inc., San Diego, CA, USA).

Sequencing libraries were constructed using the NEBNext^®^ Ultra™ RNA Library Prep Kit for Illumina^®^ (NEB, Ipswich, MA, USA), in accordance with the manufacturer’s recommendations. Index codes were added to link the generated sequences to their corresponding samples. mRNA was purified from total RNA using poly-T oligo-attached magnetic beads. Fragmentation was conducted using divalent cations under elevated temperature in NEB NextFirst^®^ Strand Synthesis Reaction Buffer (5×). First-strand cDNA was synthesized using random hexamer primer and M-MuLV Reverse Transcriptase (RNase H, NEB). Second-strand cDNA synthesis was conducted using DNA Polymerase I and RNase H. The remaining overhangs were subjected to conversion into blunt ends through the action of exonuclease/polymerase activities (NEB). Following adenylation of 3′ ends of DNA fragments, NEBNext Adaptors with a hairpin loop structure were ligated in preparation for hybridization. Library fragments were purified using the AMPure XP system (Beckman Coulter, Beverly, MA, USA) to preferentially select cDNA fragments of 250–300 bp in length. Subsequently, 3 µL of USER Enzyme (NEB) was employed with size-selected, adaptor-ligated cDNA at 37 °C for 15 min, followed by a 5 min incubation at 95 °C prior to PCR. Subsequently, PCR was conducted with Phusion High-Fidelity DNA polymerase, Universal PCR primers, and Index (X) Primer. Finally, PCR products were purified using the AMPure XP system (Beckman Coulter) and the quality of the library was evaluated using the Agilent Bioanalyzer 2100 system (Agilent Technologies, Palo Alto, CA, USA).

### 2.8. Host Transcriptome Assembly and Functional Annotation

Raw Illumina reads were subjected to a series of trimming operations, aimed at removing adapter sequences, low-quality positions (with a Phred quality score of 20 serving as the threshold), and reads containing poly-N and those shorter than 50 bp. The resulting high-quality (HQ) reads were deposited at the NCBI SRA Database and employed in the subsequent downstream analyses. As no reference genome is available for *H. diversicolor supertexta*, de novo transcriptome assembly was conducted using the Trinity assembler (v.2.4.0), with the min_kmer_cov set to 2 and all other parameters set to their default values [29,30]. The obtained unigenes were functionally annotated by searching for similar hits in seven databases: Nr (NCBI non-redundant protein sequences, e-value = 1 × 10^−5^), Nt (NCBI non-redundant nucleotide sequences, e-value = 1 × 10^−5^), KO (KEGG Orthology database, e-value = 1 × 10^−10^), GO (Gene Ontology, e-value = 1 × 10^−6^), KOG (euKaryotic Ortholog Groups, e-value = 1 × 10^−3^), Pfam (Protein family, e-value = 1 × 10^−2^), and Swiss-Prot (e-value = 1 × 10^−5^).

### 2.9. Analysis of Differentially Expressed Genes (DEGs)

Differential expression analysis was conducted on two groups using the DESeq R package version 1.10.1 (R Core Team, Vienna, Austria). DESeq provides statistical procedures for identifying differential expression in digital gene expression data using a negative binomial distribution model. The resulting *p* values were adjusted using the Benjamini and Hochberg approach for controlling the false discovery rate. The *p* value was adjusted using the *q* value. The threshold for significantly differential expression was set at *q* value < 0.05 & log_2_ (fold change) > 0.5 [31]. The statistical enrichment of differential expression genes in KEGG pathways was tested using KOBAS software [32]. Gene expression heatmaps with hierarchical clustering of expression profiles were created with the pheatmap package in R software version 4.3.2 (R Core Team, Vienna, Austria).

### 2.10. Validation of Differential Gene Expression by RT-qPCR

To validate the gene expression values obtained from the transcriptomic analysis, we selected nine DEGs for qRT-PCR validation. These DEGs met the following two criteria: (i) they were involved in immune or metabolism-related pathways, and (ii) exhibited high or moderate expression levels. Primers were designed using Premier 5 [33], and only those with amplification efficiencies between 95–105% were selected (Table S1). First-strand cDNA was synthesized from 2 µg of total RNA using reverse transcriptase (Takara, Tokyo, Japan) and random primers. qPCR was performed using THUNDERBIRDTM Next SYBR^®^ qPCR Mix (TOYOBO, Osaka, Japan) on the CFX Connect™ Real-Time System (Bio-Rad Laboratories, Inc., Hercules, CA, USA). The thermal cycling conditions were as follows: pre-denaturation at 95 °C for 30 s, followed by 40 cycles of 95 °C for 10 s and 61 °C for 33 s, and a melt curve step (starting at 65 °C, gradually increasing by 0.5 °C/s to 95 °C). Gene expression levels between samples were normalized using *Cytc1* as the internal reference gene [27]. All reactions were performed in triplicate, and the expression values were calculated as the mean relative expression using the 2^−△△CT^ method [34].

## 3. Results

### 3.1. Infection of HAHV-1 in Suspended and Adherent Primary Hemocytes

**Figure 1:**
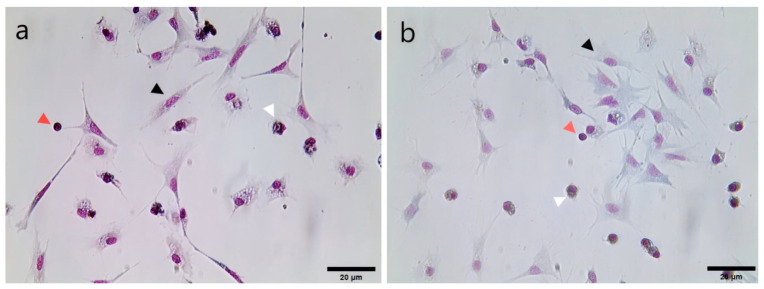
Morphological observation of hemocytes in two species of abalone by Giemsa staining: (**a**) *H. diversicolor supertexta* hemocytes; (**b**) *H. discus hannai* hemocytes. Black arrowheads indicate granulocytes, white arrowheads indicate hyalinocytes, and red arrowheads indicate blast-like cells.

In the present study, Giemsa staining was initially employed on the hemocytes of *H. diversicolor supertexta* and *H. discus hannai* in adherent cultures, which revealed three distinct morphological types of hemocytes from both species under a light microscope. The first type is granulocytes, which are predominantly irregularly oval and characterized by a large, light purple cytoplasm containing dark purple granules (Figure 1). The second type is hyalinocytes, which are subrounded or spindle-shaped, with pseudopods of varying lengths extending from the cell’s periphery. The nucleus is variable in shape, including round and oval, while the cytoplasm is small, light purple, and devoid of granules (Figure 1). The third type is blast-like cells, which are predominantly round and have the darkest-stained nucleus among the three types. The dark purple nucleus occupies most of the cell, while the cytoplasm is minimal and difficult to observe (Figure 1).

**Figure 2:**
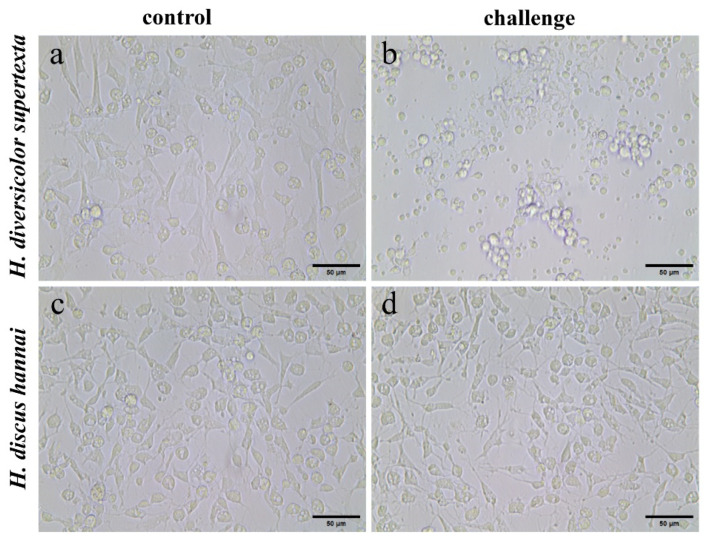
Morphologic features of abalone hemocytes challenged with HAHV-1 virus. (**a**,**b**) *H. diversicolor supertexta* hemocyte control group and challenge group; (**c**,**d**) *H. discus hannai* hemocyte control group and challenge group.

In the subsequent experiment, HAHV-1 was inoculated into primary hemocytes from both adherent and suspension cultures of two abalone species to assess the infectivity of HAHV-1 under both culture conditions. In the adherent culture group, HAHV-1 challenged *H. diversicolor supertexta* hemocytes exhibited significant shrinkage and aggregation at 24 hpi compared to the control group. The cells ruptured and dispersed, and many hemocytes were unable to maintain their adherence. The results suggest that all three types of hemocytes exhibited cytopathic effects, indicating that all three cell types may have been susceptible to HAHV-1 infection (Figure 2c,d). In contrast, the *H. discus hannai* hemocyte morphology challenge group exhibited no substantial alterations when compared to the control group (Figure 2a,b).

**Figure 3:**
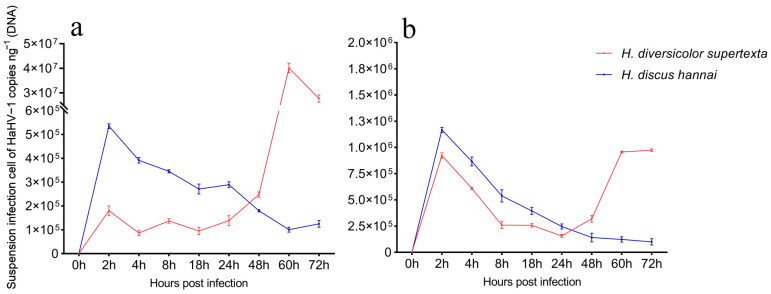
The change in HAHV-1 copies in infected hemocytes and hemolymph under *in vitro* conditions. (**a**) The change in HAHV-1 copies of *H. diversicolor supertexta* and *H. discus hannai* hemocytes, respectively; (**b**) The change in HAHV-1 copies of *H. diversicolor supertexta and H. discus hannai* hemolymph, respectively. The error bars indicate the standard deviation (SD) of the mean of HAHV-1 DNA load.

In the suspension culture group, the data indicated that viral copies in *H. diversicolor supertexta* hemocytes increased during the initial two hours of infection. Between 2 and 18 hpi, a decline in viral loads was observed. At 18 hpi, a recurrent increase in viral DNA copies was observed. As the infection progressed, the viral copies continued to rise, ultimately reaching a peak of 4.0 × 10⁷ copies/ng of total DNA. In contrast, in the *H. discus hannai* hemocyte group, viral copies exhibited a rapid increase during the initial 2 h inoculation period, followed by a decline as the infection progressed. The observed trend of viral copy variation in the hemolymph of both groups exhibited a high degree of similarity to that observed in the hemocytes (Figure 3).

### 3.2. Transcriptome Assembly and Functional Annotation

The generation of 12 cDNA libraries resulted in a total of 660 M of clean reads, with 168 M, 184 M, 137 M, and 173 M reads from the ZN24 (24 h negative control), ZP24 (24 hpi), ZP60 (60 hpi), and ZP60 (60 h negative control) groups, respectively (Table 1). The transcriptome was de novo assembled to generate 1.6 × 10^5^ unigenes with an average length of 1071 bp and an N50 of 1628 bp. Of these unigenes, 6.18 × 10^4^ (38.5%) were between 200 and 500 bp in length, 5.56 × 10^4^ (34.6%) were between 500 and 1000 bp in length, 2.42 × 10^4^ (15.1%) were between 1000 and 2000 bp in length, and 1.89 × 10^4^ (11.8%) were longer than 2 kbp in length (Figure S1).

We annotated the assembled unigenes with the Nr, NT, KO, Swiss-prot, Pfam, GO, and KOG databases for analysis. A total of 107,657 unigenes (67.08%) were annotated in at least one database, and 1635 unigenes (1.01%) were annotated in all seven databases (Table S2). In the homology search of the Nr database, 19.3% of the unigenes had the highest agreement with *Nephila clavipes*, followed by *Lottia gigantea* (8.0%), *Hyalella azteca* (7%), *Mizuhopecten yessoensis* (6.1%), *Aplysia californica* (4.2%), and other species (55.5%) (Table S3).

To determine the potential functions of the *H. diversicolor supertexta* transcriptome, the assembled unigenes were aligned and analyzed against the GO, KOG, and KEGG databases. A total of 58,340 (36.35%) unigenes were annotated in the GO database and classified into 56 functional subcategories under the three GO categories (biological process, cellular component and molecular function) (Table S4). A large number of immune-related genes were enriched under ‘immune system processes’ (388, 0.67%) and ‘stimulus response’ (8835, 15.14%).

The results of KOG functional annotation are shown in Table S5; 19,133 unigenes (11.92%) were classified into 26 functional families, and a few (108, 0.56%) were assigned to defense mechanisms. The results of KEGG functional annotation are shown in Table S6; 3415 unigenes were assigned to 32 KEGG pathways. Similar to the GO and KOG annotations, the KEGG pathway analysis revealed 16 immune-related pathways containing 106 unigenes.

### 3.3. DEG Expression

**Figure 4:**
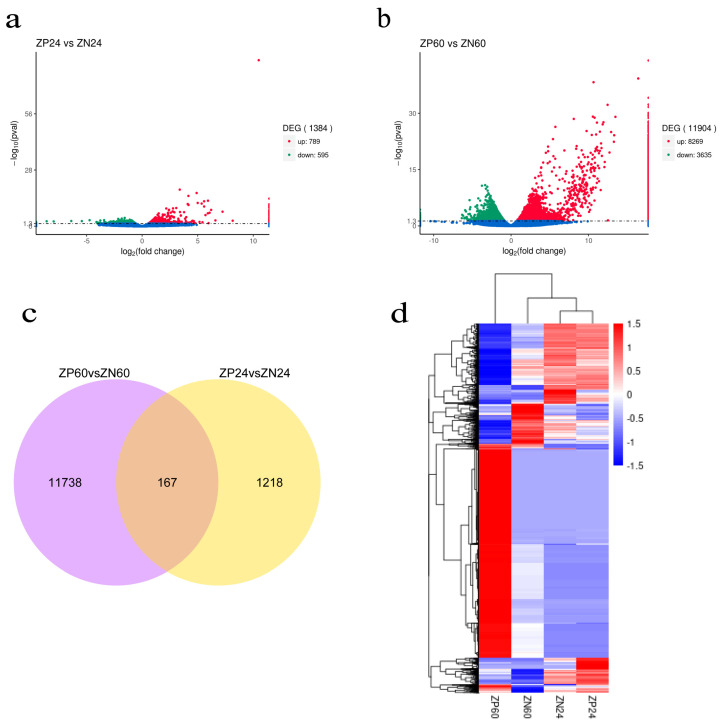
Volcano, Venn plots and heatmap of DEGs. (**a**) Volcano plots of DEGs comparing ZP24 and ZN24 groups; (**b**) Volcano plots of DEGs comparing P60 and N60 h groups; the red spots in panels (**a**,**b**) depict over-DEGs, the green spots depict under-DEGs, whereas the blue spots refer to non-differentially expressed genes; (**c**) Venn plots of DEGs among the different comparisons; (**d**) DEG expression clustering. DEG expression values are represented through the color scale from blue to red.

Based on the differential expression analysis (log_2_ fold change > 0.5, adjust *p*-value < 0.05), 1384 significant DEGs were identified between ZP24 and ZN24. These DEGs include 789 up-regulated unigenes and 595 down-regulated unigenes (Figure 4a). Furthermore, a total of 11,904 significant DEGs were identified between ZP60 and ZN60, including 8269 up-regulated unigenes and 3635 down-regulated unigenes (Figure 4b). The Venn diagram showed that a total of 13,933 DEGs were differentially expressed, of which 167 DEGs were differentially expressed at both 24 and 60 hpi (Figure 4c, Table S7), including *LAMC1* and *SRC*; *LAMC1* is involved in the PI3K-Akt signaling pathway and ECM-receptor interaction, while *SRC* is involved in endocytosis, the chemokine signaling pathway, and focal adhesion. These signaling pathways are related to the host’s immune response to the virus and may play a crucial role in the immune response to the virus in *H. diversicolor supertexta*. DEG clustering distribution was visualized in a heatmap (Figure 4d). This finding indicates that there is a greater degree of transcriptome alteration during the later stages of *in vitro* infection, compared to the earlier stages, with significant reprogramming observed in the later stages of infection.

### 3.4. GO Term and KEGG Pathway Analysis

**Figure 5:**
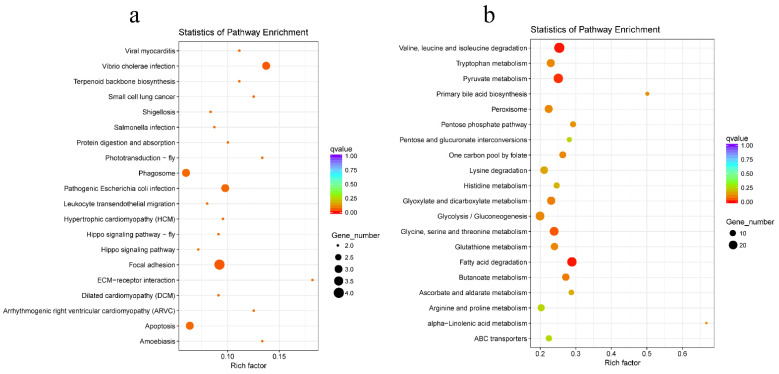
Scatter plot of KEGG enrichment analysis of DEGs. (**a**) Top 20 down-regulated KEGG pathways between ZP24 and ZN24. (**b**) Top 20 up-regulated KEGG pathways between ZP60 and ZP24. The larger the Rich factor, the greater the degree of enrichment. The value range of the *q* value is (0,1). The closer it is to zero, the more significant the enrichment is. Pathways with *q* ≤ 0.05 are defined as pathways that are significantly enriched in DEGs.

The investigation revealed a substantial up-regulation of calcium ion binding and odorant binding between ZP24 and ZN24 (Table S8). Conversely, KEGG pathway enrichment analysis revealed no pathways with a *p*-value less than 0.05 that were significantly up-regulated. However, a large number of pathways were found to be down-regulated (Table S9). The top 20 most down-regulated pathway terms are displayed in Figure 5a, with numerous pathways linked to immune responses (e.g., focal adhesion, apoptosis, and phagosome), pathogen invasion (e.g., *Vibrio cholerae* infection, pathogenic *Escherichia coli* infection, and amoebiasis) or disease processes (e.g., small cell lung cancer, arrhythmogenic right ventricular cardiomyopathy (ARVC), and viral myocarditis). This suggests a deficiency in the immune response during the early infection phase. The down-regulated KEGG pathways and related genes are shown in Table 2. Of particular note is the identification of the actin-related gene *ACTB_G1* (log_2_ foldchange (FC) = −1.03) in the majority of the down-regulated signaling pathways, followed by the laminin-related gene *LAMC1* (log_2_ FC = −2.64) and the collagen-related gene *COL4A* (log_2_ FC = −0.89). Additionally, the tubulin-related gene *TUBA* (log_2_ FC = −1.29) and the protein disulfide isomerase A4-related gene *PDIA4* (log_2_ FC = −0.76) were also identified.

Between ZP60 and ZN60, GO enrichment analysis reveals significant up-regulation of the metabolic process and catalytic activity (Table S10). KEGG pathway enrichment analysis yielded comparable results (Table S11); the top 20 most up-regulated KEGG pathways between ZP60 and ZN60 were predominantly associated with metabolic activities (Figure 5b),. These include the amino acid metabolism (e.g., valine, leucine and isoleucine degradation, pyruvate metabolism and glycine, serine and threonine metabolism), lipid metabolism (fatty acid degradation) and glucose metabolism (glycolysis/gluconeogenesis). Additionally, at 60 hpi, up-regulation of antioxidant-related genes was observed, including *SOD2* (Log_2_ FC = 9.89) and *CAT* (Log_2_ FC = 1.55).

### 3.5. Validation of Expression Data by RT-qPCR Validation

The results of qRT-PCR revealed that the expression patterns of the selected DEGs were consistent with the trends observed in the RNA-seq results. The relative expression values from both qRT-PCR and RNA-seq are presented as log_2_ (fold change) for the selected genes. The correlation between the expression levels of qRT-PCR and RNA-seq is shown in Figure S2.

## 4. Discussion

In this study, an *in vitro* HAHV-1 infection model was established using primary suspension cultures of abalone hemocytes. The study identified the varying susceptibilities of hemocytes from *H. diversicolor supertexta* and *H. discus hannai in vitro*. Subsequent to this, transcriptomic analysis was performed on HAHV-1-sensitive *H. diversicolor supertexta* hemocytes at the early and late stages of infection. This analysis revealed HAHV-1′s immune evasion during the early phase and the host’s metabolic reprogramming during the later phase.

In mollusks, hemocytes are the primary immune effector cells, playing a crucial immunological role in defending against foreign pathogen invasion in invertebrates [22,23,24]. The classification of molluscan hemocytes can be performed in several ways. Firstly, a morphological classification can be made according to the presence or absence of cellular granules and cell size. Secondly, a classification can be made using flow cytometry, transmission electron microscopy observation of hemocyte ultrastructure, and determination of the karyoplasmic ratio [24,25,35,36,37]. In the present study, the hemocytes of *H. diversicolor supertexta* and *H. discus hannai* were classified into three primary types: granulocytes, hyalinocytes, and blast-like cells. This classification system was previously employed by Choi et al. in their study of *H. diversicolor supertexta*, yielding analogous results [38]. The results of our challenge experiment indicated that all three types of hemocytes in *H. diversicolor supertexta* appeared to be susceptible to HAHV-1 infection, while *H. discus hannai* hemocytes showed limited susceptibility to HAHV-1. This finding is consistent with the observed difference in susceptibility to *in vivo* infection between the two species of abalone [1]. It is noteworthy that HAHV-1 and Ostreid herpesvirus1 (OsHV-1) are currently the only two known herpesviruses in mollusks, and phylogenetic analysis shows that they are closely related [11]. Morga et al. employed the same suspension culture method to challenge *C. gigas* hemocytes with OsHV-1 and observed a rapid increase in OsHV-1 loads at the early stages of infection, reaching a peak at approximately 18 h [26]. In contrast, the rapid increase in HAHV-1 loads observed in our study occurred at the late stage of infection, reaching a peak at approximately 60 hpi. In a related study, Zhang et al. exposed primary *Chlamys farreri* hemocytes cultured in adherent culture to OsHV-1. As observed in our experiment, the challenged group exhibited notable cytopathic effects compared to the control group [19]. In summary, *H. diversicolor supertexta* hemocytes, as immune effector cells, can support HAHV-1 replication under *in vitro*, making them an ideal model for studying HAHV-1.

It has been demonstrated that viruses are capable of manipulating the actin cytoskeleton and microtubule network in order to suppress the host’s immune response [39,40]. A substantial body of research has been conducted on the vertebrate immune response [41,42,43,44], with actin and tubulin being fundamental structural components of the actin cytoskeleton and microtubule network, respectively. These components have been shown to be crucial in the host’s immune response to viral infections [39,45]. Actin is a key component of various immune-related processes, including interferon release [46], phagocytosis [47], granule release [48,49], apoptosis [50], immune cell migration, and immune synapse formation [51,52,53]. Tubulin, on the other hand, plays a regulatory role in the innate immune system by modulating the transport of immune factors, regulating the localization of signaling intermediates that activate antiviral gene expression, and promoting autophagy [40,45]. In the present study, the *in vitro* inoculation of *H. diversicolor supertexta* hemocytes exhibited a distinct early immune deficiency. This finding is consistent with the delayed immune response observed in previous *in vivo* HAHV-1 infection studies, in which Bai et al. [27] hypothesized that HAHV-1 primarily achieves immune escape through a cell-to-cell mode. A similar inefficient immune response has also been observed during the pre-infection period following OsHV-1 infection in susceptible *C. gigas* families. The resistant *C. gigas* families exhibited an efficient immune response early in infection, with significant up-regulation of various immune-related genes, including antiviral effectors and apoptosis-related proteins, thereby effectively controlling viral replication [54]. In our study, transcriptome analysis revealed the down-regulation of *ACTB_G1* and *TUBA*, which were enriched in several down-regulated immune-signaling pathways such as focal adhesion and apoptosis. The down-regulation of these genes may be indicative of the viral manipulation of the host’s actin cytoskeleton and microtubule network, leading to the disruption of innate immune processes such as pathogen recognition, pathogen phagocytosis, and apoptosis. It is noteworthy that immune suppression phenomena mediated by the same mechanisms have also been observed in other viruses. For instance, HIV has been reported to induce remodeling changes in host cell actin, leading to CD4+ lymphocytes exhibiting severe impairment of chemotaxis and immune synapse formation, and aberrant enhancement of actin-dependent structures is observed in myeloid cells. Alterations in actin remodeling also facilitate viral dissemination via actin-dependent cell–cell mode [51,53,55]. Furthermore, HIV has been observed to hijack the microtubule network, thereby facilitating immune evasion and replication [40]. Consequently, we hypothesize that the absence of an early immune response is a consequence of the combined effects of viral manipulation of the host cell’s actin cytoskeleton and microtubule network, in conjunction with the cell-to-cell mode of transmission.

However, it is important to note that actin and tubulin also play key roles in the viral invasion process, including viral entry, replication, and release [40,43,56]. It is noteworthy that while immune responses in vertebrates and invertebrates differ significantly, the underlying biological processes of their innate immunity are fundamentally analogous, involving both the actin cytoskeleton and microtubule network. A significant role for actin and tubulin in molluscan immunity has also been demonstrated [57]. For instance, Yu et al. identified the primary interacting proteins of the putative envelope proteins ORF25 and ORF72 of OsHV-1 in *Scapharca (Anadara) broughtonii* hemocytes as actin and tubulin, respectively [13,14]. It has also been established that human papillomavirus type 16 (HPV16) utilizes laminin as an attachment receptor to facilitate viral entry [58,59]. After the virus enters the host cell, the hijacked microtubule network facilitates the transportation of complexes comprising viral genes and proteins to the replication sites, thereby promoting viral replication [40,60]. Furthermore, tunneling nanotubes (TNTs), which are actin-rich structures connecting the cytoplasm of neighboring cells, have been observed to facilitate viral spread and the infection of adjacent cells [56,61]. In the present study, HAHV-1 load increased slowly during the first 24 hpi, with a decline observed between 2 and 18 hpi. This phenomenon may be indicative of immune activity in hemocytes during the early stages, which could potentially inhibit HAHV-1 replication. Therefore, we hypothesize that the down-regulation of actin, tubulin, and laminin-related genes may reflect an indirect immune response of *H. diversicolor supertexta* hemocytes during the early stages of viral infection. This reaction may involve mechanisms, such as the reduction of virus attachment receptors, the inhibition of actin rearrangement, and the disruption of microtubule network transport. Collectively, these mechanisms contribute to the suppression of virus entry, replication, and propagation.

A substantial body of evidence indicates that viral infection results in the metabolic reprogramming of the host cell, and that the viral hijacking of the cellular metabolism is a key mechanism facilitating viral replication [62,63,64,65] and immune escape [64]. At 60 hpi, in contrast to the robust immune response observed during the later stages of *in vivo* HAHV-1 infection in *H. diversicolor supertexta* [27], the viral load in the later stages of *in vitro* hemocyte inoculation peaked without any significant immune response. The intense immune response observed in the later stages of *in vivo* HAHV-1 infection in *H. diversicolor supertexta* is inefficient, with approximately 50% mortality occurring within the subsequent 12 h. This viral response pattern is similar to that observed in the susceptible *C. gigas* families [27,54]. Instead, a significant number of metabolic pathways exhibited an increase in activity. The most notable changes include the up-regulation of glycolysis, glutamine metabolism signaling pathways, and antioxidant-related genes. HAHV-1 primarily infects two types of host tissues, nerve and hemocytes, resulting in acute death [1] and chronic death [66]. The mechanism underlying chronic mortality remains unclear. We speculate that the observed chronic mortality may result from the viral infection gradually hijacking the energy metabolism and redox metabolism of hemocytes, thereby impairing their immune capacity. This impairment is hypothesized to exacerbate the infection over time, ultimately leading to death, which aligns with the lack of immune response observed in the later stages of inoculation in our study. Lorgeril et al. reached conclusions that are similar to our own in their study of OsHV-1, concluding that OsHV-1 is able to inhibit the immune response of the oyster by invading the hemocytes of the susceptible *C. gigas*, altering hemocyte function and leading to the exacerbation of infection, and that early and effective immunity is key to disease control [54]. The enhanced glycolysis observed in the context of the Warburg effect has been demonstrated to be a pivotal factor in the rapid generation of the energy necessary for viral replication. This mechanism has been demonstrated to play a crucial role in the invasion of various viruses, including OsHV-1 and white spot syndrome virus (WSSV) [67,68]. It has been demonstrated that viral infection of host cells results in the increased utilization and metabolism of glutamine, thereby providing ATP and precursors for macromolecule synthesis during viral replication [69]. In addition, the up-regulation of glutamine metabolism and antioxidant genes has been shown to reduce intracellular oxidative stress, thereby creating a favorable cellular environment for viral replication [64]. The majority of virus-induced metabolic reprogramming has been observed to enhance fatty acid synthesis, thereby providing sufficient substrates for viral replication [70]. Interestingly, in contrast to the observations made by some researchers, where viral infection promotes fatty acid synthesis, the present study found that fatty acid degradation was markedly induced in host cells during the late stage of HAHV-1 inoculation. This warrants further in-depth investigation in the future [71,72].

The present transcriptome analysis identifies substantial changes in immune-related and metabolic pathways during infection. However, the reliance on transcript-level changes without proteomics or functional assays to verify gene function may limit the biological relevance of these findings. Moreover, the present results were obtained from an *in vitro* infection model under controlled conditions, and the lack of *in vivo* correlation limits the applicability of the results to natural infections. It is therefore recommended that future studies focus on both *in vitro* and *in vivo* approaches, and further validate the results through proteomics and functional assays, thereby providing a more comprehensive understanding of the interactions between HAHV-1 and the host.

## 5. Conclusions

In this study, we confirmed that *H. diversicolor supertexta* suspension cultures can serve as an *in vitro* infection model to investigate the interaction mechanisms between HAHV-1 and the host. Our transcriptomic analysis suggests that early immune deficiency may result from HAHV-1’s manipulation of the host’s actin cytoskeleton and microtubule network. Furthermore, we propose that the rapid increase in viral copies during the later stages of infection is due to the virus hijacking the host’s energy and redox metabolism to promote its own replication. In summary, our results provide a reference for the establishment of an *in vitro* infection platform for HAHV-1, and inform breeding programs for HAHV-1-resistant abalone species and optimal management strategies.

## Figures and Tables

**Table 1:** Summary of the sequencing and assembly results.

Group	Sample	Raw Reads	Clean Reads	Clean Bases	Error (%)	Q20 (%)	Q30 (%)	GC (%)
ZN24	ZN24–1	55,658,664	54,940,790	8.24 G	0.03	97.38	92.87	45.11
	ZN24–2	59,667,454	58,936,052	8.84 G	0.03	97.09	92.22	45
	ZN24–3	53,519,200	52,924,078	7.94 G	0.03	97.29	92.68	44.85
ZP24	ZP24–1	62,335,812	61,503,080	9.23 G	0.03	97.39	92.95	44.9
	ZP24–2	60,602,778	59,854,832	8.98 G	0.03	97.26	92.64	44.92
	ZP24–3	63,585,518	62,763,338	9.41 G	0.03	97.47	93.1	44.58
ZN60	ZN60–1	46,913,992	44,993,012	6.75 G	0.03	95.4	89.21	41.5
	ZN60–2	51,417,304	47,316,656	7.1 G	0.03	95.2	88.68	41.75
	ZN60–3	45,533,266	44,246,870	6.64 G	0.03	95.29	88.65	42.01
ZP60	ZP60–1	57,023,124	55,994,658	8.4 G	0.03	95.7	89.31	42.15
	ZP60–2	64,805,608	63,376,048	9.51 G	0.03	95.85	89.63	42.24
	ZP60–3	55,118,306	53,781,690	8.07 G	0.03	95.67	89.28	42.3

**Table 2:** Down-regulated pathways and related *genes* in *H. diversicolor supertexta* hemocytes at 24 hpi.

Pathway	ID	*q* Value	Down-Regulated Genes	
			Name	Entry
Focal adhesion	Ko04510	0.048	*ACTB_G1*	K05692
			*LAMC1*	K05635
			*COL4A*	K06237
Vibrio cholerae infection	Ko05110	0.048	*ACTB_G1*	K05692
			*PDIA4*	K09582
Pathogenic Escherichia coli infection	Ko05130	0.069	*ACTB_G1*	K05692
			*TUBA*	K07374
Amoebiasis	Ko05146	0.069	*LAMC1*	K05635
			*COL4A*	K06237
Small cell lung cancer	Ko05222	0.069	*LAMC1*	K05635
			*COL4A*	K06237
Arrhythmogenic right ventricular cardiomyopathy (ARVC)	ko05412	0.069	*ACTB_G1*	K05692
Apoptosis	ko04210	0.069	*ACTB_G1*	K05692
			*TUBA*	K07374
Viral myocarditis	ko05416	0.069	*ACTB_G1*	K05692
Phagosome	ko04145	0.069	*ACTB_G1*	K05692
			*TUBA*	K07374

## Data Availability

All the raw data discussed in this paper have been submitted to the NCBI SRA database under accession ID PRJNA1214030.

## Supplementary Materials

The following supporting information can be downloaded at: https://www.mdpi.com/article/10.3390/biology14020121/s1. Figure S1: Length distribution of assembled unigenes; Figure S2: Verification of transcriptomic DEGs by qRT-PCR; Table S1. Primers used for qPCR validation of RNA-seq results; Table S2: Annotation success rate statistics of unigenes in various databases; Table S3: Functional annotation of the assembled unigenes; Table S4: GO annotation of the *Haliotis diversicolor supertexta* transcriptome; Table S5: KOG annotation of the *Haliotis diversicolor supertexta* transcriptome; Table S6: KEGG annotation of the *Haliotis diversicolor supertexta* transcriptome; Table S7: List of differentially expressed genes (DEGs) with annotations and expression values; Table S8: GO annotation for up-regulated DEGs between ZP24 and ZN24 groups; Table S9: KEGG pathway assignment down-regulated DEGs between ZP24 and ZN24 groups; Table S10: GO annotation for up-regulated DEGs between ZP60 and ZN60 groups; Table S11: KEGG pathway assignment up-regulated DEGs between ZP60 and ZN60 groups.

## References

[1] Bai C.M., Li Y.N., Chang P.H., Jiang J.Z., Xin L.S., Li C., Wang J.Y., Wang C.M. (2018). Susceptibility of two abalone species, *Haliotis diversicolor supertexta* and *Haliotis discus hannai*, to Haliotid herpesvirus 1 infection. J. Invertebr. Pathol..

[2] Wang J.Y., Guo Z.X., Feng J., Liu G.F., Xu L.W., Chen B.S., Pan J.P. (2004). Virus infection in cultured abalone, *Haliotis diversicolor Reeve* in Guangdong Province, China. J. Shellfish Res..

[3] Gu L., Qi R.J., Yang R., Han T., Jiang J.Z., Wang J.Y. (2019). The prevalence of abalone herpesvirus in two *Haliotis* species in South China during 2002–2013. Aquaculture.

[4] Hooper C., Hardy-Smith P., Handlinger J. (2007). Ganglioneuritis causing high mortalities in farmed Australian abalone (*Haliotis laevigata* and *Haliotis rubra*). Aust. Vet. J..

[5] Chang P.H., Kuo S.T., Lai S.H., Yang H.S., Ting Y.Y., Hsu C.L., Hon C.C. (2005). Herpes-like virus infection causing mortality of cultured abalone Haliotis diversicolor supertexta in Taiwan. Dis. Aquat. Organ..

[6] Corbeil S., Williams L.M., McColl K.A., Crane M.S.J. (2016). Australian abalone (Haliotis laevigata, *H. rubra* and *H. conicopora*) are susceptible to infection by multiple abalone herpesvirus genotypes. Dis. Aquat. Organ..

[7] Corbeil S., McColl K.A., Williams L.M., Mohammad I., Hyatt A.D., Crameri S.G., Fegan M., Mark S.J.C. (2012). Abalone viral ganglioneuritis: Establishment and use of an experimental immersion challenge system for the study of abalone herpes virus infections in Australian abalone. Virus Res..

[8] Hooper C., Slocombe R., Day R., Crawford S. (2012). Leucopenia associated with abalone viral ganglioneuritis. Aust. Vet. J..

[9] Davison A.J., Trus B.L., Cheng N.Q., Steven A.C., Watson M.S., Cunningham C., Le Deuff R.M., Tristan R. (2005). A novel class of herpesvirus with bivalve hosts. J. Gen. Virol..

[10] He Y., Jouaux A., Ford S.E., Lelong C., Sourdaine P., Mathieu M., Guo X.M. (2015). Transcriptome analysis reveals strong and complex antiviral response in a mollusc. Fish Shellfish Immun..

[11] Savin K.W., Cocks B.G., Wong F., Sawbridge T., Cogan N., Savage D., Simone W. (2010). A neurotropic herpesvirus infecting the gastropod, abalone, shares ancestry with oyster herpesvirus and a herpesvirus associated with the amphioxus genome. Virol. J..

[12] Green T.J., Peter S. (2018). Antiviral defense and innate immune memory in the oyster. Viruses.

[13] Martenot C., Faury N., Morga B., Degremont L., Lamy J.B., Houssin M., Tristan R. (2019). Exploring first interactions between Ostreid Herpesvirus 1 (OsHV-1) and its host, *Crassostrea gigas*: Effects of specific antiviral antibodies and dextran sulfate. Front. Microbiol..

[14] Yu J.N., Liu Y., Huang B., Li C., Wang D.D., Yao M.L., Xin L.S., Bai C.M., Wang C.M. (2021). Characterization of host cell potential proteins interacting with OsHV-1 membrane proteins. Viruses.

[15] Potts R.W.A., Gutierrez A.P., Cortés-Araya Y., Houston R.D., Tim P.B. (2020). Developments in marine invertebrate primary culture reveal novel cell morphologies in the model bivalve *Crassostrea gigas*. Peer J..

[16] Yoshino T.P., Bickham U., Bayne C.J. (2013). Molluscan cells in culture: Primary cell cultures and cell lines. Can. J. Zool..

[17] Potts R.W.A., Regan T., Ross S., Bateman K., Hooper C., Paley R., Houston R.D., Bean T.P. (2024). Laboratory replication of Ostreid Herpes Virus (OsHV-1) using pacific oyster tissue explants. Viruses.

[18] van der Merwe M., Auzoux-Bordenave S., Niesler C., Roodt-Wilding R. (2010). Investigating the establishment of primary cell culture from different abalone (*Haliotis midae*) tissues. Cytotechnology.

[19] Ji A., Li X., Fang S., Qin Z., Bai C., Wang C., Zhang Z. (2017). Primary culture of Zhikong scallop Chlamys farreri hemocytes as an *in vitro* model for studying host-pathogen interactions. Dis. Aquat. Organ..

[20] Hanana H., Talarmin H., Pennec J.P., Droguet M., Gobin E., Marcorelle P., Dorange G. (2011). Establishment of functional primary cultures of heart cells from the clam *Ruditapes decussatus*. Cytotechnology.

[21] Li Y., Jiang S., Li M., Xin L., Wang L., Wang H., Qiu L., Song L. (2016). A cytokine-like factor astakine accelerates the hemocyte production in Pacific oyster *Crassostrea gigas*. Dev. Comp. Immunol..

[22] Allam B., Raftos D. (2015). Immune responses to infectious diseases in bivalves. J. Invertebr. Pathol..

[23] Chu F.L.E., Fingerman N.R. (2000). Defense mechanisms of marine bivalves. Recent Advances in Marine Biotechnology. Immunobiology and Pathology.

[24] Wang W., Li M., Wang L., Chen H., Liu Z., Jia Z., Qiu L., Song L. (2017). The granulocytes are the main immunocompetent hemocytes in Crassostrea gigas. Dev. Comp. Immunol..

[25] Estrada N., Velázquez E., Rodríguez-Jaramillo C., Ascencio F. (2013). Morphofunctional study of hemocytes from lions-paw scallop *Nodipecten subnodosus*. Immunobiology.

[26] Morga B., Faury N., Guesdon S., Chollet B., Renault T. (2017). Haemocytes from *Crassostrea gigas* and OsHV-1: A promising *in vitro* system to study host/virus interactions. J. Invertebr. Pathol..

[27] Bai C.M., Zhang S.M., Li Y.N., Xin L.S., Rosani U., Wang C.M. (2019). Dual transcriptomic analysis reveals a delayed antiviral response of *Haliotis diversicolor supertexta* against Haliotid herpesvirus-1. Viruses.

[28] Schikorski D., Faury N., Pepin J.F., Saulnier D., Tourbiez D., Renault T. (2011). Experimental ostreid herpesvirus 1 infection of the Pacific oyster *Crassostrea Gigas*: Kinetics of virus DNA detection by q-PCR in seawater and in oyster samples. Virus Res..

[29] Bai C.M., Rosani U., Li Y.N., Zhang S.M., Xin L.S., Wang C.M. (2019). RNA-seq of HaHV-1-infected abalones reveals a common transcriptional signature of Malacoherpesviruses. Sci. Rep..

[30] Grabherr M.G., Haas B.J., Yassour M., Levin J.Z., Thompson D.A., Amit I., Adiconis X., Fan L., Raychowdhury R., Zeng Q. (2011). Full-length transcriptome assembly from RNA-Seq data without a reference genome. Nat Biotechnol..

[31] Love M.I., Huber W., Anders S. (2014). Moderated estimation of fold change and dispersion for RNA-seq data with DESeq2. Genome Biol..

[32] Mao X., Cai T., Olyarchuk J.G., Wei L. (2005). Automated genome annotation and pathway identification using the KEGG Orthology (KO) as a controlled vocabulary. Bioinformatics.

[33] Singh V.K., Mangalam A.K., Dwivedi S., Naik S. (1998). Primer premier: Program for design of degenerate primers from a protein sequence. BioTechniques.

[34] Livak K.J., Schmittgen T.D. (2001). Analysis of relative gene expression data using real-time quantitative PCR and the 2^−ΔΔCT^ method. Methods.

[35] Zhou L., Yang A., Liu Z., Wu B., Sun X., Lv Z., Tian J., Du M. (2017). Changes in hemolymph characteristics of ark shell *Scapharaca broughtonii* dealt with *Vibrio anguillarum* challenge *in vivo* and various of anticoagulants *in vitro*. Fish Shellfish Immunol..

[36] Karetin Y.A., Kalitnik A.A., Safonova A.E., Cicinskas E. (2019). Description and classification of bivalve mollusks hemocytes: A computational approach. Peer J..

[37] Rolton A., Delisle L., Berry J., Venter L., Webb S.C., Adams S., Hilton Z. (2020). Flow cytometric characterization of hemocytes of the flat oyster, *Ostrea chilensis*. Fish Shellfish Immunol..

[38] Hong H.K., Donaghy L., Choi K.S. (2019). Flow cytometric characterization of hemocytes of the abalone *Haliotis diversicolor* (Reeve, 1846) and effects of air exposure stresses on hemocyte parameters. Aquaculture.

[39] Taylor M.P., Koyuncu O.O., Enquist L.W. (2011). Subversion of the actin cytoskeleton during viral infection. Nat. Rev. Microbiol..

[40] Seo D., Gammon D.B. (2022). Manipulation of host microtubule networks by viral microtubule-associated proteins. Viruses.

[41] Guo H., Shen S., Wang L., Deng H. (2010). Role of tegument proteins in herpesvirus assembly and egress. Protein Cell.

[42] Madavaraju K., Koganti R., Volety I., Yadavalli T., Shukla D. (2021). Herpes simplex virus cell entry mechanisms: An update. Front. Cell. Infect. Microbiol..

[43] Connolly S.A., Jackson J.O., Jardetzky T.S., Longnecker R. (2011). Fusing structure and function: A structural view of the herpesvirus entry machinery. Nat Rev Microbiol.

[44] Horst D., Ressing M.E., Wiertz E.J.H.J. (2011). Exploiting human herpesvirus immune evasion for therapeutic gain: Potential and pitfalls. Immunol Cell Biol.

[45] Martín-Cófreces N.B., Sánchez-Madrid F. (2018). Sailing to and docking at the immune synapse: Role of tubulin dynamics and molecular motors. Front. Immunol..

[46] Jans J., elMoussaoui H., De Groot R., De Jonge M.I., Ferwerda G. (2016). Actin- and clathrin-dependent mechanisms regulate interferon gamma release after stimulation of human immune cells with respiratory syncytial virus. Virol. J..

[47] Martins R., Maier J., Gorki A.D., Huber K.V.M., Sharif O., Starkl P., Saluzzo S., Quattrone F., Gawish R., Lakovits K. (2016). Heme drives hemolysis-induced susceptibility to infection via disruption of phagocyte functions. Nat. Immunol..

[48] Ritter A.T., Kapnick S.M., Murugesan S., Schwartzberg P.L., Griffiths G.M., Lippincott-Schwartz J. (2017). Cortical actin recovery at the immunological synapse leads to termination of lytic granule secretion in cytotoxic T lymphocytes. Proc. Natl. Acad. Sci. USA.

[49] Carisey A.F., Mace E.M., Saeed M.B., Davis D.M., Orange J.S. (2018). Nanoscale dynamism of actin enables secretory function in cytolytic cells. Curr Biol..

[50] Li F., Fan X., Zhang Y., Zhang Y., Ma X., Kou J., Yu B. (2018). Inhibition of myosin IIA–actin interaction prevents ischemia/reperfusion induced cardiomyocytes apoptosis through modulating PINK1/Parkin pathway and mitochondrial fission. Int. J. Cardiol..

[51] Gordón-Alonso M., Sala-Valdés M., Rocha-Perugini V., Pérez-Hernández D., López-Martín S., Ursa A., Álvarez S., Kolesnikova T.V., Vázquez J., Sánchez-Madrid F. (2012). EWI-2 association with α-actinin regulates T cell immune synapses and HIV viral infection. J. Immunol..

[52] Irving A.T., Wang D., Vasilevski O., Latchoumanin O., Kozer N., Clayton A.H.A., Szczepny A., Morimoto H., Xu D., Williams B.R.G. (2012). Regulation of actin dynamics by protein kinase R control of gelsolin enforces basal innate immune defense. Immunity.

[53] Ospina Stella A., Turville S. (2018). All-round manipulation of the actin cytoskeleton by HIV. Viruses.

[54] de Lorgeril J., Lucasson A., Petton B., Toulza E., Montagnani C., Clerissi C., Vidal-Dupiol J., Chaparro C., Galinier R., Escoubas J.-M. (2018). Immune-suppression by OsHV-1 viral infection causes fatal bacteraemia in Pacific oysters. Nat. Commun..

[55] Anand A.R., Zhao H., Nagaraja T., Robinson L.A., Ganju R.K. (2013). N-terminal slit2 inhibits HIV-1 replication by regulating the actin cytoskeleton. Retrovirology.

[56] Lv W., Li Z., Wang S., He J., Zhang L. (2024). A role for tunneling nanotubes in virus spread. Front. Microbiol..

[57] Ye T., Tang W., Zhang X. (2012). Involvement of Rab6 in the regulation of phagocytosis against virus infection in invertebrates. J. Proteome Res..

[58] Richards K.F., Mukherjee S., Bienkowska-Haba M., Pang J., Sapp M. (2014). Human papillomavirus species-specific interaction with the basement membrane-resident non-heparan sulfate receptor. Viruses.

[59] Stavolone L., Lionetti V. (2017). Extracellular matrix in plants and animals: Hooks and locks for viruses. Front. Microbiol..

[60] Dharan A., Campbell E.M. (2018). Role of microtubules and microtubule-associated proteins in HIV-1 infection. J. Virol..

[61] Ganti K., Han J., Manicassamy B., Lowen A.C. (2021). Rab11a mediates cell-cell spread and reassortment of influenza a virus genomes via tunneling nanotubes. PLoS Pathog..

[62] Thyrsted J., Christian K.H. (2021). Virus-induced metabolic reprogramming and innate sensing hereof by the infected host. Curr. Opin. Biotechnol..

[63] Thaker S.K., Ch’ng J., Christofk H.R. (2019). Viral hijacking of cellular metabolism. BMC Biol..

[64] Li J., Wang Y., Deng H., Li S., Qiu H.-J. (2023). Cellular metabolism hijacked by viruses for immunoevasion: Potential antiviral targets. Front. Immunol..

[65] Moreno-Altamirano M.M.B., Kolstoe S.E., Sánchez-García F.J. (2019). Virus control of cell metabolism for replication and evasion of host immune responses. Front. Cell. Infect. Microbiol..

[66] Chen I.-W., Chang P.H., Chen M.-S., Chen M.-S., Renault T., Chen M.-M., Kuo S.T., Cheng C. (2016). Exploring the chronic mortality affecting abalones in taiwan: Differentiation of abalone herpesvirus-associated acute infection from chronic mortality by pcr and in situ hybridization and histopathology. Taiwan Vet. J..

[67] Corporeau C., Tamayo D., Pernet F., Quéré C., Madec S. (2014). Proteomic signatures of the oyster metabolic response to herpesvirus OsHV-1 μVar infection. J. Proteomics.

[68] Su M.A., Huang Y.T., Chen I.T., Lee D.Y., Hsieh Y.C., Li C.Y., Ng T.H., Liang S.Y., Lin S.Y., Huang S.W. (2014). An invertebrate warburg effect: A shrimp virus achieves successful replication by altering the host metabolome via the PI3K-Akt-mTOR pathway. PLoS Pathog..

[69] Hirabara S.M., Gorjao R., Levada-Pires A.C., Masi L.N., Hatanaka E., Cury-Boaventura M.F., Da Silva E.B., dos Santos-Oliveira L.C., Sousa Diniz V.L., Serdan T.A.D. (2021). Host cell glutamine metabolism as a potential antiviral target. Clin. Sci..

[70] Shen T., Wang T. (2021). Metabolic reprogramming in COVID-19. Int. J. Mol. Sci..

[71] Bappy S.S., Haque Asim M.M., Ahasan M.M., Ahsan A., Sultana S., Khanam R., Shibly A.Z., Kabir Y. (2024). Virus-induced host cell metabolic alteration. Rev. Med. Virol..

[72] Mayer K.A., Stöckl J., Zlabinger G.J., Gualdoni G.A. (2019). Hijacking the supplies: Metabolism as a novel facet of virus-host interaction. Front. Immunol..

