# Supplementary figures and images for: Mechanisms of HAHV-1 Interaction with Hemocytes in Haliotis diversicolor supertexta: An In Vitro Study
Source: Biology (Basel). 2025 Jan 24;14(2):121. doi: 10.3390/biology14020121 (PMC11851962; doi:10.3390/biology14020121)

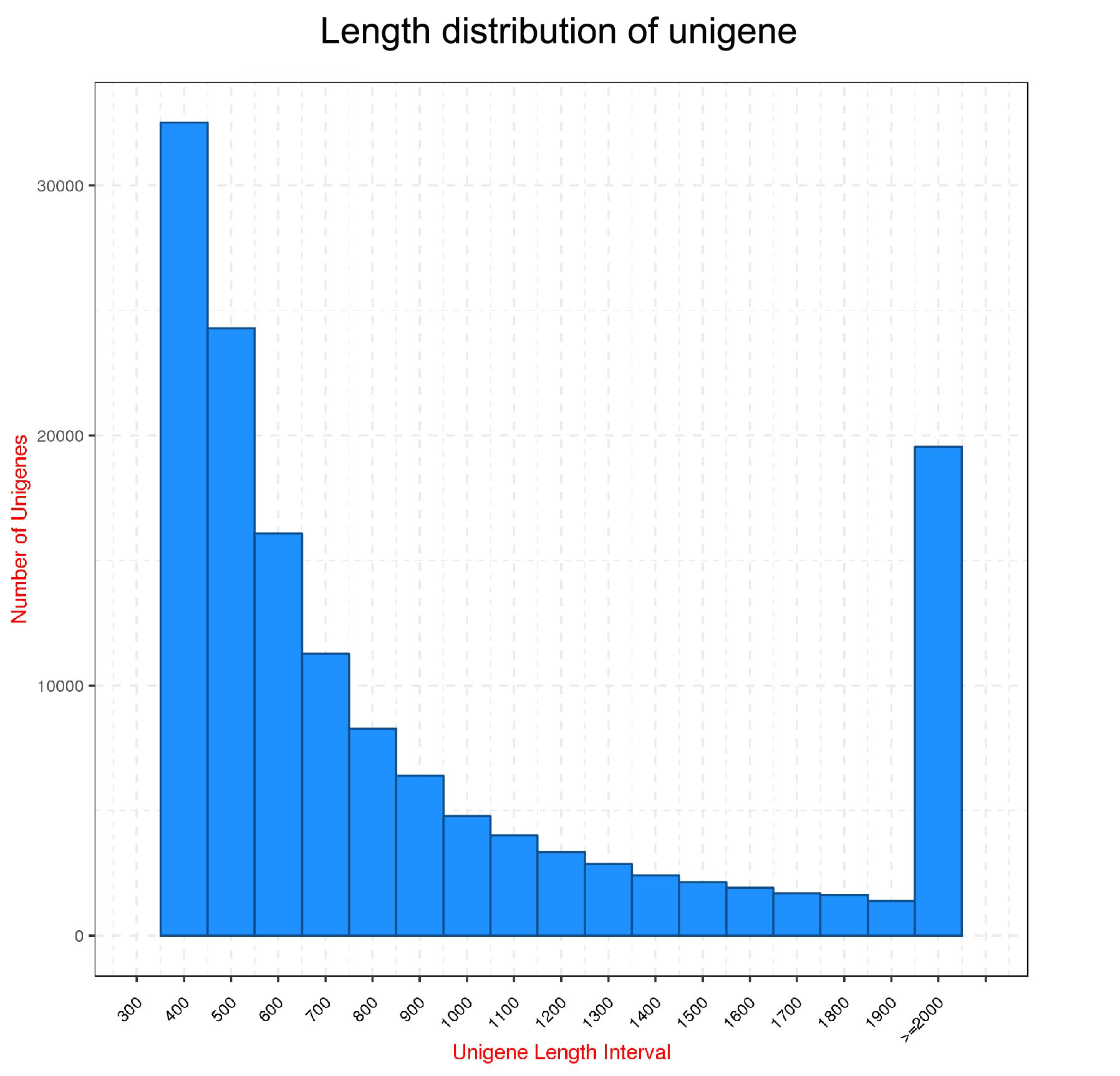

Supplement: Supplementary file 1 [file biology-14-00121-s001.zip › Figure S1.tif]

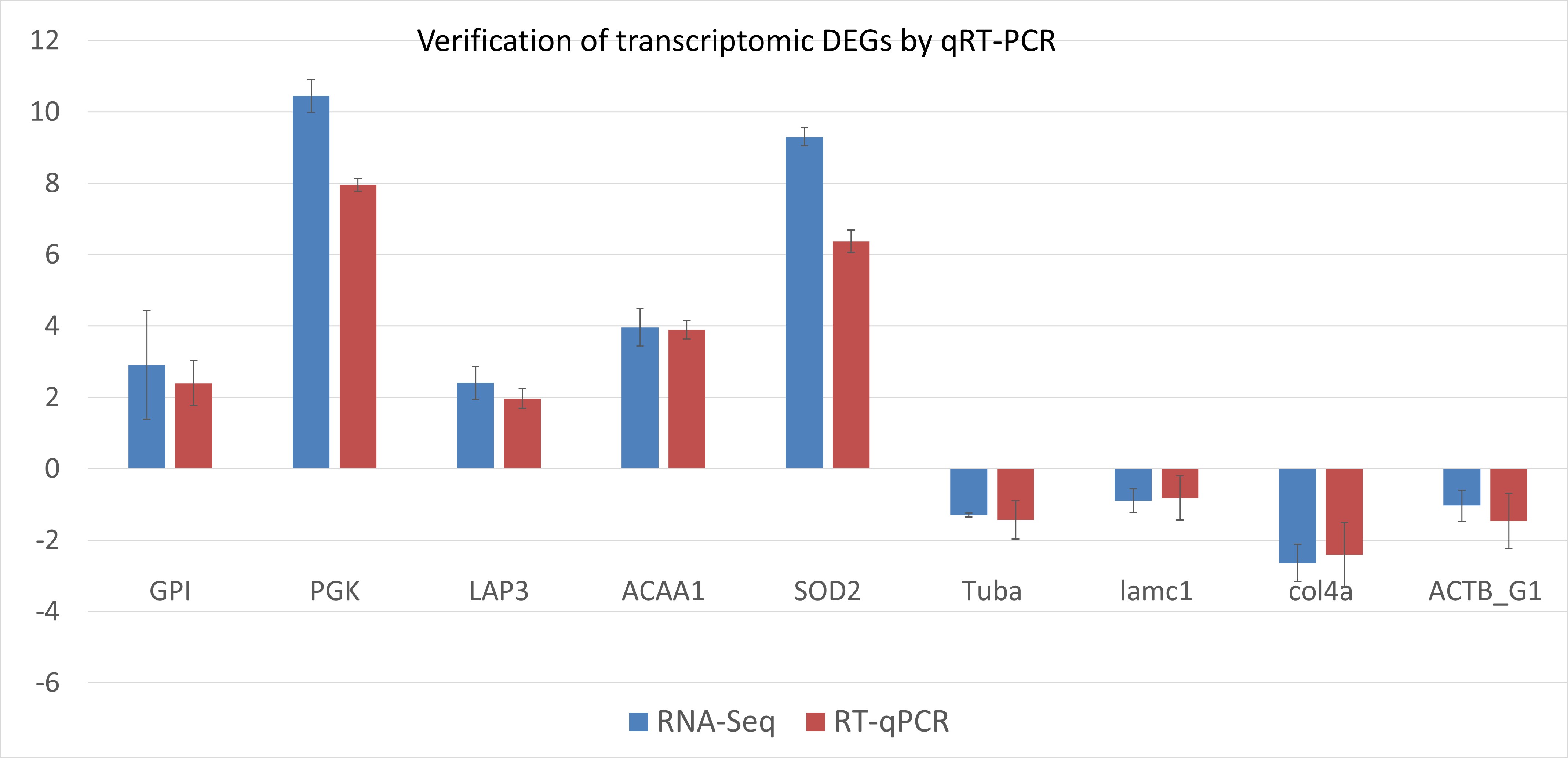

Supplement: Supplementary file 1 [file biology-14-00121-s001.zip › Figure S2..jpg]
